# Endovascular adhesive repair using N-butyl cyanoacrylate for spontaneous dissecting aneurysms of the celiac and hepatic arteries: technique and long-term outcomes

**DOI:** 10.1186/s42155-026-00768-7

**Published:** 2026-09-04

**Authors:** Hideki Ishimaru, Satomi Yoshimi, Tomoki Nakano, Shuto Miyamura, Taiga Oka, Chika Somagawa, Maki Hirao, Takahiro Kagimoto, Tomoki Minami, Ryo Toya

**Affiliations:** 1 Department of Radiological Sciences, Nagasaki University Graduate School of Biomedical Sciences, 1-7-1 Sakamoto, Nagasaki, 852-8501 Japan; 2 Department of Radiology, Nagasaki University Hospital, Nagasaki, Japan; 3 Department of Radiology, National Hospital Organization Nagasaki Medical Center, Omura, Japan

**Keywords:** Celiac artery, Hepatic artery, Dissecting aneurysm, N-butyl cyanoacrylate, Therapeutic embolization, Segmental arterial mediolysis

## Abstract

**Purpose:**

To describe false lumen embolization with an endovascular adhesive repair concept, in which N-butyl cyanoacrylate is injected with the intention of promoting reapposition of the dissected layers while preserving the parent-artery.

**Materials and methods:**

Three men aged 50, 55, and 68 years with spontaneous dissecting aneurysms of the celiac axis (two patients) or the right hepatic artery (one patient, with dissection extending from the common hepatic artery) were treated; one patient had segmental arterial mediolysis; in another, the gastroduodenal collateral pathway had been surgically interrupted by prior pancreaticoduodenectomy. Indications were aneurysm enlargement with persistent pain, progressive aneurysm enlargement, and concern for rupture during planned endoscopic treatment. A microcatheter was navigated into the false lumen under digital subtraction angiography, and a 33% N-butyl cyanoacrylate–iodized oil mixture was injected to embolize the false lumen and appose the intimal flap. In two patients, a silicone balloon catheter was used to limit N-butyl cyanoacrylate overflow into the true lumen; it was introduced through a separate femoral access in Case 1 and through the same guiding sheath as the microcatheter in Case 2. Technical and clinical success, parent-artery patency, complications, and recurrence were assessed.

**Results:**

Evident inflow into the false lumen disappeared in all cases, with all parent-arteries preserved. There was no rupture, unintended non-target embolization, or catheter adhesion. Follow-up showed no recurrence, and patency was verified during clinically indicated vascular studies at 10.5, 6, and 4 years; in two patients the repaired segment again served as a catheterization route.

**Conclusion:**

False lumen embolization with an endovascular adhesive repair concept was technically successful with parent-artery preservation in three highly selected patients, and patency was confirmed at 10.5, 6, and 4 years after treatment. Further investigation is required to determine the safety, reproducibility, and appropriate indications of this technique.

**Level of evidence:**

Level 4, Case Series.

**Supplementary Information:**

The online version contains supplementary material available at https://doi.org/10.1186/s42155-026-00768-7.

## Introduction

Spontaneous celiac artery dissection is uncommon, and no consensus management algorithm exists [1]. Some cases arise from segmental arterial mediolysis, a nonatherosclerotic arteriopathy producing visceral dissection and aneurysm [2].

Endovascular options have limitations. Coil isolation sacrifices the parent-artery [3], whereas covered stent-grafts preserve flow but are hard to deliver through the tortuous celiac axis [4]. In aortic surgery, adhesives such as gelatin-resorcin-formalin glue reinforce the dissected layers [5, 6], suggesting the principle could be applied endovascularly within the false lumen.

Coils are the usual embolic agent for visceral lesions, with N-butyl cyanoacrylate used when a microcatheter is too small for coils or coils fail to occlude the target [7]. In balloon remodeling, a balloon is temporarily inflated across the neck during coil or liquid-embolic delivery, then deflated and removed, restoring distal flow [8]; experimentally, a balloon can also appose and glue dissected layers [9]. We combined these principles into an endovascular adhesive repair and report our technique and outcomes in three patients.

## Materials and methods

### Patients

Three men were treated for a spontaneous dissecting aneurysm of the celiac axis (Cases 1 and 2) or right hepatic artery (Case 3; Table 1). Case 1 presented with persistent abdominal pain during follow-up for multiple hepatocellular carcinomas. No other abnormality explaining the pain was identified, and the dissecting aneurysm enlarged concurrently; the pain resolved after treatment. Case 2 was asymptomatic and had segmental arterial mediolysis diagnosed on imaging 5 years earlier, without pathological confirmation, with dissecting aneurysms of the celiac, superior mesenteric, and left gastric arteries; the false lumen contained both entry and re-entry. Case 3 had no symptoms attributable to the hepatic arterial dissection. He had a prior aortic dissection (abdominal 7 years earlier, thoracic 4 months earlier) and a previous pancreaticoduodenectomy that had interrupted the gastroduodenal collateral pathway. CT obtained when he presented with choledocholithiasis demonstrated intramural hematoma extending from the common to the right hepatic artery. Angiography showed a pinhole entry (< 1 mm) without a re-entry and a caudate branch arising from the false lumen. Because mechanical stimulation during endoscopic treatment of choledocholithiasis was considered to carry a risk of rupture, the hepatic arterial dissection was treated first. Given the prior aortic dissections, classification as a spontaneous visceral artery dissection should be interpreted cautiously, although the hepatic arterial lesion was remote in time from the abdominal aortic dissection and anatomically separate from the thoracic aortic dissection.

**Table 1:** Patient, lesion, procedural characteristics, and outcomes

Variable	Case 1	Case 2	Case 3
Patient and clinical course			
Age/Sex	50/M	55/M	68/M
Background	Multiple HCCs	SAM (celiac, SMA, LGA dissections)	Prior aortic dissections; prior pancreaticoduodenectomy
Presentation and course	Abdominal pain; enlargement over 3 days; resolved after treatment	Asymptomatic; incidental 5 years earlier; gradual enlargement	Asymptomatic; found on CT during choledocholithiasis workup; IMH, common–right HA
Indication for treatment	Enlarging aneurysm + persistent pain	Progressive enlargement	Feared rupture during planned endoscopic stone removal
Lesion morphology			
Treated parent artery	Celiac	Celiac	Right HA
False-lumen diameter	7.0 mm	6.0 mm	8.0 mm
True-lumen diameter	3.0 mm	3.8 mm	2.0 mm
Maximum overall diameter	10.0 mm	9.8 mm	10.0 mm
Dissection length	18.0 mm	19.0 mm	15.0 mm
Entry	2.0 mm	1.6 mm	Pinhole, < 1 mm
Re-entry	None	1.7 mm	None
Branch from false lumen	None	None	Caudate branch
Procedure			
Access	Bilateral femoral	Right femoral	Right femoral
Guiding sheath/catheter	5.3-Fr and 4-Fr shepherd-hook catheters	6-Fr Flexor Ansel guiding sheath	7-Fr Flexor Ansel guiding sheath
Balloon catheter	Masamune silicone	Masamune silicone	None
Microcatheter	Carnelian PIXIE 1.8-Fr	Excelsior SL-10	Progreat Lambda 1.7-Fr
Catheter configuration	Balloon + microcatheter, separate accesses	Balloon + microcatheter, parallel via one 6-Fr sheath	Microcatheter via guiding sheath
Balloon position	Across entry	At re-entry; entry left open	Not used
NBCA–iodized oil	33%; ≤ 1.5 mL*	33%; ≤ 1.5 mL*	33%; ≤ 1.5 mL*
Other dissected segments	None	LGA (coils); SMA observed	LHA embolized; CHA observed (IMH, no entry)
Outcome			
Outcome	Success; patent at 1 week and 10.5 years; no recurrence	Success; patent at 1 week and 6 years; no recurrence	Success; patent at 1 week and 4 years; no recurrence

*HCC* hepatocellular carcinoma, *SAM* segmental arterial mediolysis, *SMA* superior mesenteric artery, *LGA* left gastric artery, *HA* hepatic artery, *LHA* left hepatic artery, *CHA* common hepatic artery, *IMH* intramural hematoma, *NBCA* N-butyl cyanoacrylate, *y* years, *wk* week. Morphologic measurements were obtained immediately before treatment. The initial diameter for Case 1 could not be measured because the computed tomogram at initial presentation was no longer available. *Injected volumes were not recorded retrospectively; procedural records confirmed the total NBCA–iodized oil mixture did not exceed 1.5 mL in any case

### Technique

All procedures were performed under local anesthesia via a femoral approach, without intraprocedural heparinization. In Case 1, the balloon catheter and microcatheter were introduced through separate femoral accesses. In Case 2, both were introduced in parallel through a single 6-Fr Flexor Ansel guiding sheath via one right femoral puncture. After angiographic identification of the entry and re-entry, the microcatheter was positioned within the false lumen, beyond the entry, in a stable position, without withdrawing it during injection. In Cases 1 and 2, a silicone balloon catheter (Masamune; Fuji Systems Corporation, Tokyo, Japan) was inflated with 50% diluted iodinated contrast medium before the microcatheter was flushed with glucose solution. The balloon covered the entry in Case 1. In Case 2, the available balloon was too short to cover both the entry and the re-entry; it was therefore placed at the re-entry, and the entry remained open and was monitored continuously under DSA. A 33% (1:2) mixture of N-butyl cyanoacrylate (Histoacryl; B. Braun, Melsungen, Germany) and iodized oil (Lipiodol; Guerbet, Villepinte, France) was injected under DSA for approximately 30 s or less. Injection was stopped when the false lumen appeared filled and before any reflux toward the entry occurred; because N-butyl cyanoacrylate expands slightly on polymerization, injection was kept deliberately conservative. Exact case-specific volumes and injection rates were not recorded; the total mixture volume did not exceed 1.5 mL in any case. The microcatheter was withdrawn promptly, and balloon deflation was initiated immediately without an intentional waiting interval and completed gradually over approximately 1 min. In Case 3, a balloon was not used because the entry was < 1 mm and reflux was considered unlikely. No dedicated bailout device was prepared; limited NBCA protrusion into the true lumen was considered acceptable provided antegrade parent-artery flow remained preserved. Dissected branches that could be sacrificed hemodynamically were embolized with coils and/or N-butyl cyanoacrylate, whereas dissections that were not enlarging were left untreated (Table 1). None of the patients was receiving antiplatelet or anticoagulant therapy at presentation, and none was added for the procedure or afterward because no stent was implanted.

### Outcome measures

Technical success was defined as preserved antegrade parent-artery flow with disappearance of evident inflow into the false lumen on completion angiography. Clinical success was defined as resolution or absence of symptoms, stabilization or resolution of the treated vascular abnormality, and freedom from recurrence or reintervention. Parent-artery patency, complications, and recurrence were assessed on follow-up computed tomographic angiography and/or angiography.

## Results

In all three cases, evident inflow into the false lumen disappeared immediately after the procedure, and all parent-arteries were preserved (Table 1). There was no intraprocedural rupture, unintended non-target embolization, or catheter adhesion; in Case 3 a caudate branch arising from the false lumen was also filled, without clinical sequelae.

**Fig. 1:**
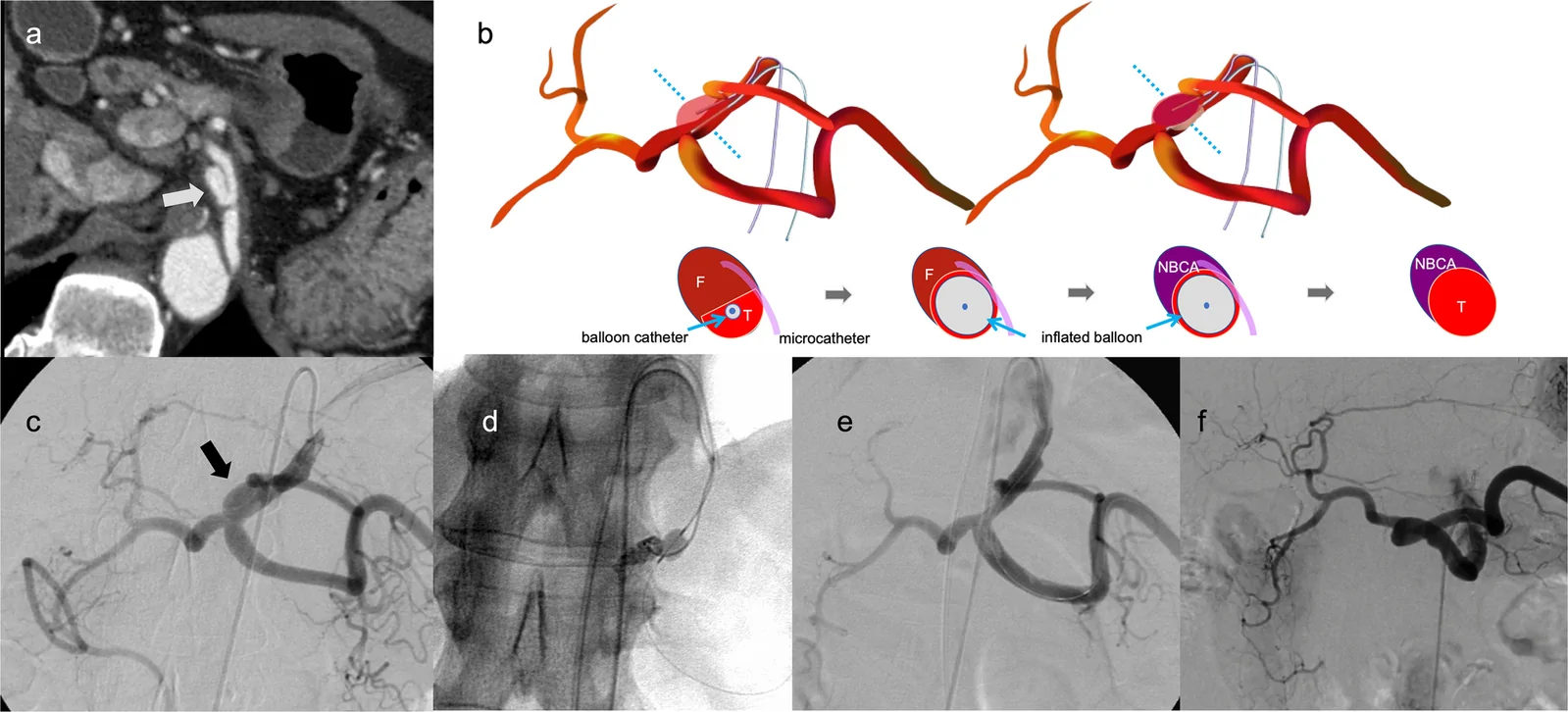
A 50-year-old man with a spontaneous dissecting aneurysm of the celiac artery (Case 1). **a** Contrast-enhanced computed tomography shows the dissecting aneurysm of the celiac artery (arrow). Multiple well-differentiated hepatocellular carcinomas, which did not enhance in the arterial phase, were present in the liver (not shown). **b** Schematic of the procedure: a microcatheter (light purple) was advanced through the entry into the false lumen (F), and a balloon catheter was placed in the true lumen; the balloon was inflated to expand the true lumen (T) while NBCA was injected into the false lumen (dark purple). The dashed line indicates the level of the cross-sections shown below, which are arranged from left to right as the injection proceeds and end with the balloon deflated and flow restored in the true lumen (T). **c** Celiac arteriography demonstrates the dissecting aneurysm (arrow). **d** Injection of the mixture into the false lumen, with the true lumen supported by the inflated balloon. **e** Celiac arteriography immediately after treatment shows disappearance of false-lumen opacification. **f** Celiac arteriography 10.5 years later, during transarterial chemoembolization for hepatocellular carcinoma. Although the projection differs from that at treatment (cranial view), the celiac artery shows no stenosis and no residual false lumen

**Fig. 2:**
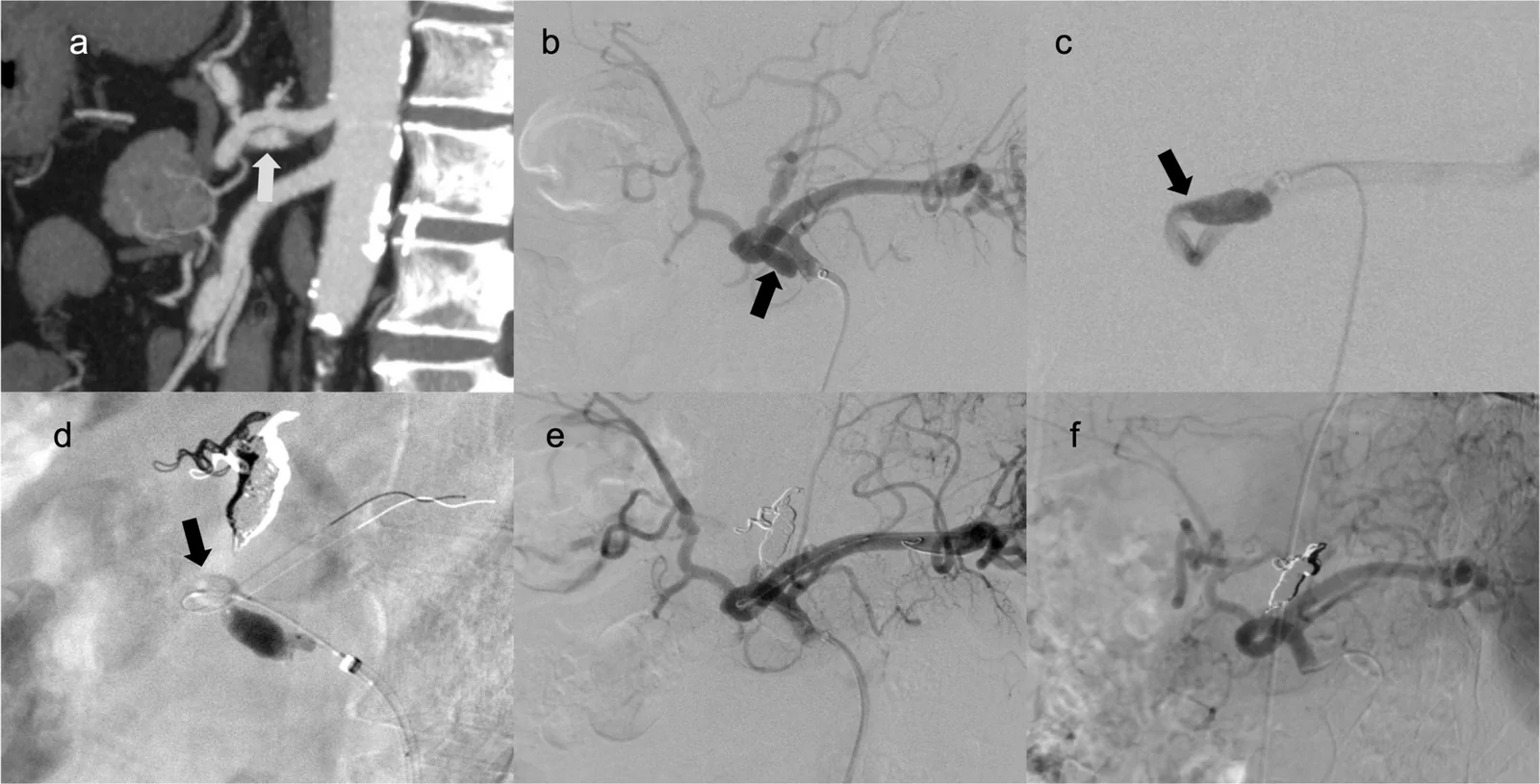
A 55-year-old man with segmental arterial mediolysis (Case 2). **a** Sagittal reformatted contrast-enhanced computed tomography shows a dissecting aneurysm of the celiac artery (arrow), together with dissecting aneurysms of the left gastric artery and the main trunk of the superior mesenteric artery. **b** Celiac arteriography shows the false lumen of the dissecting aneurysm (arrow). **c** Manual contrast injection through a microcatheter positioned in the false lumen shows contrast outflow through the re-entry (arrow) into the splenic artery. **d** Injection of the N-butyl cyanoacrylate–iodized oil mixture into the false lumen, with a balloon inflated within the true lumen against the re-entry (arrow). A video of the NBCA injection is provided in Supplementary material 1. Coil embolization of the dissecting aneurysm of the left gastric artery had been performed before this procedure. **e** Celiac arteriography immediately after treatment shows disappearance of false-lumen opacification. **f** Celiac arteriography 6 years later, obtained during a selective arterial secretin injection test, shows no stenosis of the celiac artery and no residual false lumen

**Fig. 3:**
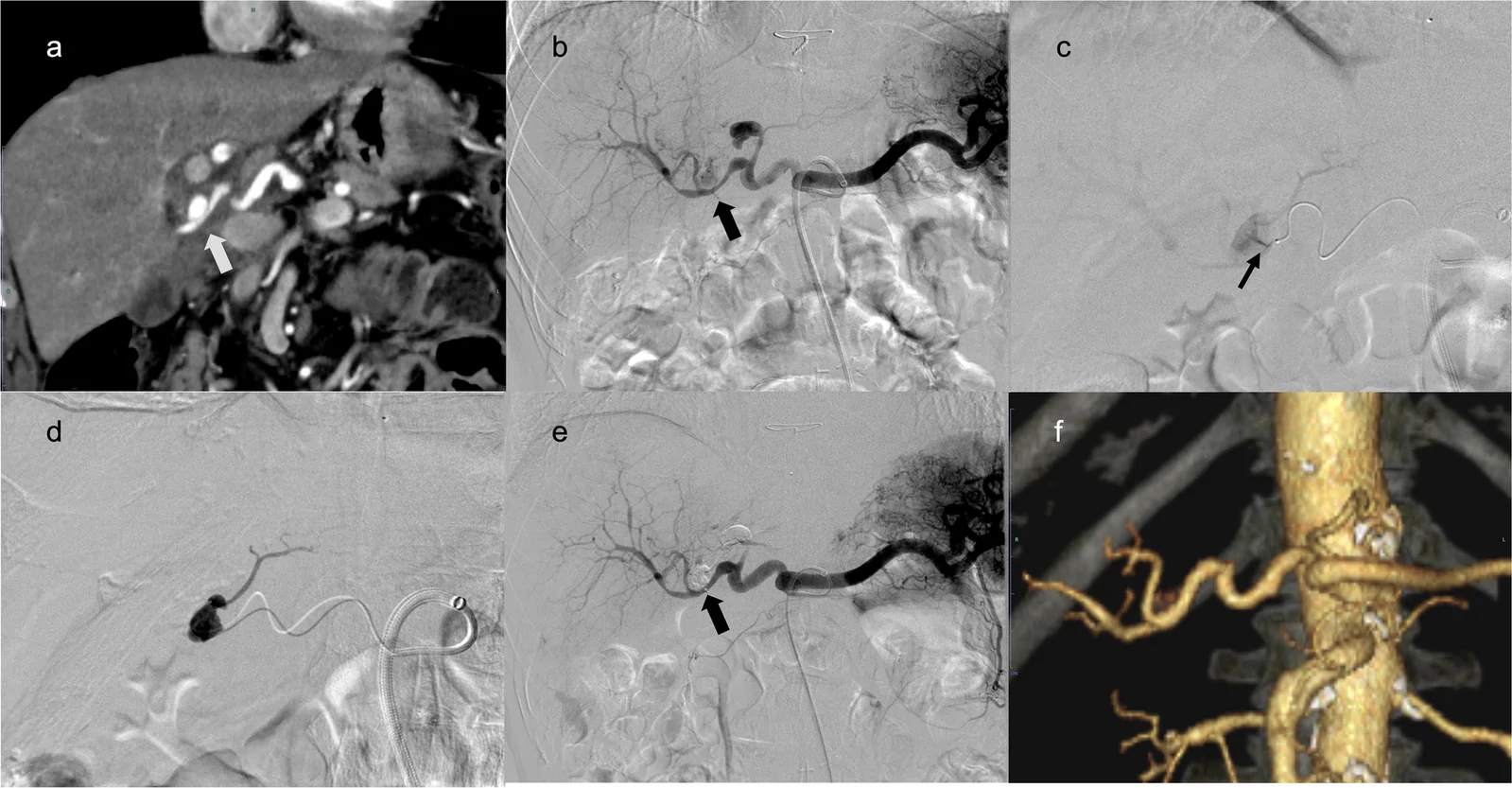
A 68-year-old man with a dissection extending from the common hepatic artery to the right and left hepatic arteries (Case 3). **a** Coronal reformatted contrast-enhanced computed tomography shows a dissecting aneurysm of the right hepatic artery (arrow). Soft-tissue attenuation surrounding the common hepatic artery suggests an intramural hematoma corresponding to a non-opacified false lumen. A dissecting aneurysm of the left hepatic artery is also visible. **b** Celiac arteriography shows the false lumen of the right hepatic arterial dissecting aneurysm (arrow), a dissecting aneurysm of the left hepatic artery, and smooth stenosis of the right hepatic artery, presumably caused by the intramural hematoma. **c** Contrast injection through a microcatheter positioned immediately proximal to the entry shows jet-like contrast inflow through the entry (arrow), clearly demonstrating its pinhole configuration. A caudate branch arising from the false lumen is also faintly opacified. **d** The microcatheter was advanced through the pinhole entry into the false lumen, followed by injection of the N-butyl cyanoacrylate–iodized oil mixture. No balloon was used because the entry was extremely small and NBCA reflux into the true lumen was considered unlikely. NBCA also entered a caudate branch arising from the false lumen. **e** Celiac arteriography immediately after treatment shows disappearance of false-lumen opacification. The dissecting aneurysm of the left hepatic artery had also been embolized with NBCA. **f** Three-dimensional volume-rendered image from contrast-enhanced computed tomography obtained 4 years later for surveillance of the thoracic and abdominal aortic dissections shows resolution of the dissecting aneurysms and disappearance of the hepatic arterial stenosis

Contrast-enhanced CT obtained approximately 1 week after treatment in each patient confirmed preserved parent-artery flow without recurrent dissection. No standardized surveillance specifically for the treated dissection was performed thereafter. The repaired segment was subsequently evaluated during clinically indicated examinations for other conditions: celiac arteriography at 10.5 years in Case 1 during chemoembolization for a new hepatocellular carcinoma (Fig. 1), arteriography at 6 years in Case 2 during a selective arterial secretin injection test (Fig. 2), and computed tomographic angiography at 4 years in Case 3 during aortic surveillance (Fig. 3). The repaired segment was patent without recurrent dissection at each examination. Cases 2 and 3 remain recurrence-free; Case 1 was followed until death from hepatocellular carcinoma progression, unrelated to the treated artery.

## Discussion

This series describes false lumen embolization with an endovascular adhesive repair concept using diluted N-butyl cyanoacrylate while preserving the parent-artery. Cyanoacrylate adhesive repair of dissected layers has been reported in the aorta as a surgical technique (glue aortoplasty) [5, 6], and N-butyl cyanoacrylate has been used in the visceral arteries to embolize pseudoaneurysms [7, 10]. In the present procedure, false lumen obliteration and preserved parent-artery flow were objectively demonstrated; re-apposition and adhesion of the dissected layers represent the intended mechanism and were not directly confirmed.

Spontaneous isolated celiac artery dissection usually follows a benign course and often remodels on conservative management [11, 12]; intervention is generally reserved for dissecting aneurysm, symptom persistence, enlargement, or malperfusion [11]. Each of our cases — all dissecting aneurysms — met such criteria (Table 1); in Case 3, treatment was performed because of concern for rupture during a planned endoscopic procedure. Preserving the parent-artery was then important for a distinct reason in each: future chemoembolization of the hepatocellular carcinoma (Case 1); avoiding increased flow demand on the superior mesenteric artery, which also harbored a dissection (Case 2); and maintaining hepatic perfusion after pancreaticoduodenectomy in an extensive dissection (Case 3).

Two points were important: the choice of a silicone balloon catheter, and familiarity with the flow and polymerization of diluted N-butyl cyanoacrylate, which otherwise risks non-target embolization or adhesion [7]. The 33% (1:2) N-butyl cyanoacrylate–iodized oil mixture is the concentration we use most routinely in embolization, including hemostatic procedures, and was selected for this familiarity rather than because other concentrations were unsuitable. A silicone balloon is preferable because N-butyl cyanoacrylate has low adhesivity to silicone, as noted with the same balloon catheter [13]: the balloon can press the polymerizing adhesive against the intimal flap yet be withdrawn without adhering to the hardened cast. Flap apposition by a balloon during gluing has limited experimental support [9]. What matters is preservation of antegrade parent-artery flow; limited N-butyl cyanoacrylate protrusion into the true lumen was considered acceptable provided that flow was not compromised: when both an entry and a re-entry are present, the re-entry must also be controlled. In Case 2, the balloon covered the re-entry while the entry was left open and continuously monitored for reflux, whereas with an entry alone only reflux through it matters. A balloon was unnecessary in Case 3 because the entry was narrow and such reflux unlikely. Provided the adhesive is contained, the dissected layers can be apposed.

Unlike parent-artery sacrifice, adhesive repair does not preclude future access: in two patients the repaired segment remained usable as a catheterization route.

Covered stent-grafts also preserve the parent-artery [4, 8], but spontaneous visceral artery dissection is not an approved indication for the covered stent-grafts available in our country, and we did not consider off-label stent-grafting justified in these patients. In Cases 1 and 2, in which the celiac artery was of adequate caliber, stent-graft placement might have been anatomically feasible; in Case 3 the true lumen measured only 2 mm and no suitable device was available. The acutely angulated, tortuous, and already dissected arteries were in any case poorly suited to the relatively stiff delivery systems required, a concern compounded in Case 2 by segmental arterial mediolysis affecting multiple segments. Adhesive repair leaves no implant and no continuing radial force, avoids metallic artifact, spares a landing zone, and requires no additional antithrombotic therapy. These considerations do not establish that adhesive repair is safer than covered stent-grafting.

### Limitations

This retrospective report included only three highly selected patients, used individualized techniques, and lacked a control group. Adhesion of the dissected layers could not be directly confirmed. Although contrast-enhanced CT was obtained approximately 1 week after treatment, no standardized surveillance specifically for the treated dissection was performed thereafter; later examinations were obtained for other clinical indications. Continuous patency throughout each follow-up interval therefore cannot be established. Although the false lumen was not intentionally converted into a completely closed space, conservative injection cannot eliminate the risks of false lumen pressurization, rupture, dissection extension, or extravasation, particularly in fragile arteries. Because balloon deflation was initiated immediately after microcatheter withdrawal, displacement or migration of an incompletely stabilized N-butyl cyanoacrylate cast remains a theoretical risk; the exact interval was not recorded. Exact injection rates and timing intervals were not recorded, and no dedicated bailout device was prepared. Limited NBCA protrusion into the true lumen was considered acceptable if antegrade parent-artery flow remained preserved, but the safety of this approach has not been established. The absence of complications in this small series does not establish procedural safety or superiority over covered stent-grafting. Further investigation is required to define safety, reproducibility, efficacy, ischemic risk, and appropriate indications.

## Conclusion

False lumen embolization with an endovascular adhesive repair concept was technically successful with long-term parent-artery patency in three highly selected patients. Further investigation is required to determine its safety, reproducibility, and appropriate indications relative to established reconstructive techniques such as covered stent-grafting.

## Supplementary Information

Supplementary Material 1. Case 2: digital subtraction angiography during injection of the N-butyl cyanoacrylate–iodized oil mixture into the false lumen with the balloon inflated at the re-entry site.

## Data Availability

All data generated or analysed during this study are included in this published article and its supplementary information file (Supplementary material 1).
